# Deceptions in Medicine

**Published:** 1844-12

**Authors:** 


*Deceptions in Medicine.*—Courses of lectures are now being delivered in Paris on the following subjects;—one on phrenology; two on magnetism; one on homœopathy; one on uromancy, and to cap the climax, one on deceptions in medicine.
*Journal des Connaiss. Med.*

